# Altered intestinal microbiota composition, antibiotic therapy and intestinal inflammation in children and adolescents with cystic fibrosis

**DOI:** 10.1371/journal.pone.0198457

**Published:** 2018-06-22

**Authors:** Maiara Brusco de Freitas, Emilia Addison Machado Moreira, Camila Tomio, Yara Maria Franco Moreno, Felipe Perozzo Daltoe, Eliana Barbosa, Norberto Ludwig Neto, Vittoria Buccigrossi, Alfredo Guarino

**Affiliations:** 1 Graduate Program in Nutrition, Federal University of Santa Catarina, Florianópolis, Brazil; 2 Department of Nutrition, Graduate Program in Nutrition, Federal University of Santa Catarina, Florianópolis, Brazil; 3 Department of Pathology, Federal University of Santa Catarina, Florianópolis, Brazil; 4 Joana de Gusmão Children´s Hospital, Santa Catarina, Florianópolis, Brazil; 5 Department of Translational Medical Science, Section of Pediatrics, University of Naples Federico II, Naples, Italy; Hospital for Sick Children, CANADA

## Abstract

The aim of the present study was to evaluate the effect of cystic fibrosis and antibiotic therapy on intestinal microbiota composition and intestinal inflammation in children and adolescents. A cross-sectional controlled study was conducted with 36 children and adolescents: 19 in the cystic fibrosis group (CFG) and 17 in the control group (CG) matched for age and sex. The CFG was subdivided based on the use of antibiotic therapy (CFAB group) and non-use of antibiotic therapy (CFnAB group). The following data were evaluated: colonization, antibiotic therapy, mutation, breastfeeding, use of infant formula, type of delivery, introduction of solid foods, body mass index, fecal calprotectin and intestinal microbiota composition (fluorescence *in situ* hybridization). Intestinal inflammation evaluated by fecal calprotectin was significantly higher in the CFG (median: 40.80 µg/g, IQR: 19.80–87.10, p = 0.040) and CFAB group (median: 62.95 µg/g, IQR: 21.80–136.62, p = 0.045) compared to the CG (median: 20.15 µg/g, IQR: 16.20–31.00), and the *Bacteroides*, Firmicutes, *Eubacterium rectale* and *Faecalibacterium prausnitzii* were significantly decreased (p < 0.05) in the CFG compared to the CG, whereas the bacteria *Clostridium difficile*, *Escherichia coli* and *Pseudomonas aeruginosa* were significantly increased in the CFG (p < 0.05). The main differences were found between the CG and CFAB group for *Eubacterium rectale* (p = 0.006), *Bifidobacterium* (p = 0.017), *Escherichia coli* (p = 0.030), Firmicutes (p = 0.002), *Pseudomonas aeruginosa* (p < 0.001) and *Clostridium difficile* (p = 0.006). The results of this study confirm intestinal inflammation in patients with CF, which may be related to changes in the composition of the intestinal microbiota.

## Introduction

Microbiome acquisition begins in the uterus and progresses during childhood. The composition of the microbiota is influenced by the type of delivery, feeding, exposure to antibiotics in the perinatal period and maternal physical contact [1, 2]. An increase in the diversity of the intestinal microbiota occurs beginning at birth and children one to three years of age have a pattern similar to that found in adults. However, the occurrence of infection results in a lower diversity of microbiota [3, 4]. In addition to being associated with health promotion, breast milk provides significant amounts of *Bifidobacterium* and *Lactobacillus* [5] and the ingestion of solid foods may or may not exert a positive influence on the microbiome, depending on the type of food [6].

The defect in the cystic fibrosis transmembrane regulator in patients with cystic fibrosis (CF) can have manifestations in the gastrointestinal tract, such as gastroesophageal reflux, viscous intestinal mucus due to dehydration, acidification of the medium, altered mucosal glycosylation [7], distal intestinal obstruction, inflammation, bacterial overgrowth in the small intestine, slow transit time, inflammatory bowel disease [8] and dysbiosis [9], the latter of which can trigger the overgrowth of opportunistic pathogens and contribute to the development of diseases [10, 11].

The mechanism underlying the inflammatory bowel process remains unclear, but inflammation in evidenced by the increase in inflammatory markers, such as calprotectin and rectal nitric oxide, in patients with CF [6, 12]. The use of antibiotics, such as tobramycin and erythromycin, is another factor that may influence the microbiota of the gastrointestinal tract [11]. While the ecological balance is normally restored within a few weeks after antibiotic ingestion, there is evidence that antibiotic-associated disorders may persist for a long time after treatment. Thus, prolonged exposure to antibiotics may result in the overgrowth of antibiotic-resistant bacteria [13], as *Clostridium difficile* (*C*. *difficile*), which can cause severe diarrhea and/or pseudomembranous colitis [14].

Studies have shown that patients with CF typically have decreased amounts of *Bifidobacterium* spp. [6], the *Bacteroides-Prevotella* group [12], *Clostridium* cluster XIVa [15], *Faecalibacterium prausnitzii* (*F*. *prausnitzii*) [6] and *Eubacterium rectale* (*E*. *rectale*) [6], whereas *Enterobacteriaceae* and *Clostridia* [14] are increased.

The clinical relevance of the change in intestinal microorganisms is not well established, but the microflora of the gastrointestinal tract is known to be related to metabolic functions, such as the regulation of host immune response. Thus, the frequent use of antibiotics can have a negative cumulative effect on the metabolism and immune system in this population [16].

The aim of the present study was to evaluate the effect of CF and antibiotic therapy on intestinal microbiota composition and intestinal inflammation in children and adolescents.

## Methods and subjects

### Study design

A cross-sectional study was conducted between March and December 2016 at the Joana de Gusmão Children’s Hospital in the city of Florianópolis, Brazil. This study received approval from the Human Research Ethics Committees of the hospital and the Federal University of Santa Catarina (certificate number: 48959715.2.1001.0121). The legal guardians of the children and adolescents signed a statement of informed consent.

## Subjects

The required sample size calculated by the difference in means was 18 for each group (OpenEpi^®^ program, 1:1 proportion of exposed to non-exposed, 80% power and 95% confidence level). Thus, the overall sample was composed of 36 children and adolescents. The control group (CG) was composed of 17 individuals with a median age of 3.00 years [interquartile range (IQR): 0.60–7.00 years] and cystic fibrosis group (CFG) was composed of 19 individuals with a median age of 4.00 years (IQR: 1.10–9.50 years). Moreover, the CFG was subdivided based on the use of antibiotic therapy (CFAB group) and non-use of antibiotic therapy (CFnAB group).

The inclusion criteria for the CFG were a diagnosis of CF (chloride sweat test ≥ 60 mmol/L) and clinical stability for at least 30 days prior to the data collection process [17]. The individuals in the CG were recruited from children’s care clinic of the hospital and matched to the CFG for age and sex, with a body mass index (BMI) in the ideal range and the absence of CF [17]. The following were the exclusion criteria for the CG: fever, trauma, diseases (celiac, respiratory, inflammatory, intestinal, rheumatic, psychiatric, degenerative, cardiovascular, diabetes mellitus, renal, primary or secondary immunodeficiency and glucose/lactose intolerance) and use of antibiotics, hormones, non-hormonal anti-inflammatory drugs, proton pump inhibitors, oral, inhaled and intravenous corticosteroids, ranitidine hydrochloride, immunosuppressants and antihistamines 30 days prior to the data collection. The exclusion criteria for the CFG were the same as those for the CG, except for the use of antibiotics. Patients taking antibiotics (colimycin, azithromycin, tobramycin, cefuroxime, ciprofloxacin, oxacillin and ceftazidime) for the treatment of lung disease were admitted to the study. Daily supplementation as part of the protocol was taken into account and supplement intake was determined on the basis of patient reports. The hospital medical protocol prescribed the following supplements: SourceCF^®^ vitamin supplement (Eurand Pharmaceuticals, Huntsville, AL, USA) for those aged ≥ 1 year to < 4 years, 2 mL/day; ADEK^®^ (Axcan Pharma, Birmingham, AL, USA) at a dose of one tablet/day for those aged ≥ 4 to < 10 years and two tablets/day for those aged ≥ 10 years [18]; CREON^®^ (Solvay Pharmaceuticals, GmbH, Germany) for older children and adults, who require 500 to 4000 lipase units per gram of fat ingested (mean = 1800 lipase units/g of fat) [19].

### Assessment of clinical characteristics, BMI, lung function and disease severity

Data on age, sex, date of birth, date of diagnosis of CF, colonization, antibiotic treatment and mutation were collected from the patients’ medical records. Information on breastfeeding, the use of an infant formula, type of delivery and the introduction of solid foods was based on parental reports. Anthropometric data (height and weight) were collected using the method described by Pereira [17]. BMI was classified based on the World Health Organization criteria [20] using the Anthro and AnthroPlus programs (WHO, Geneva, Switzerland). Bacteriological findings and lung function [assessed based on forced expiratory volume in one second (FEV_1_)] were determined as described by Pereira [17]. Disease severity was determined based on the Shwachman-Kulczycki (S-K) score [17].

### Analysis of intestinal inflammation

For the analysis of fecal calprotectin, feces were collected in plastic containers and delivered directly to the hospital. Intestinal inflammation was assessed based on fecal calprotectin, which was measured using the RIDASCREEN^®^ Calprotectin (R-biopharm-AG) immunoenzyme test. Samples with a calprotectin concentration of 0 to 50 µg/g were considered normal, values between 50 to 100 µg/g were considered intermediate and values higher than 100 µg/g were considered indicative of intestinal inflammation [21].

### Fluorescence *in situ* hybridization

A plastic straw measuring 4 cm in length and 3 mm in diameter was introduced 4 to 10 mm into the feces for the collection of the sample, which was placed in a 50-mL Falcon, fixed with 30 mL of Carnoy’s solution composed of ethanol, glacial acetic acid and chloroform (6/6/1, respectively) (Dinâmica Química Contemporânea Ltda^®^) for 24 hours at room temperature and then kept refrigerated at 4°C until analysis [22].

Fecal samples fixed in Carnoy’s solution were embedded in paraffin, cut longitudinally into 4-µm sections, placed on SuperFrost slides (*Thermo Scientific*, Italy) and dewaxed with xylene and ethanol (VWR Chemicals, United Kingdom). The samples were then incubated with a hybridization solution (20 Mm Tris-HCL, 0.9 M NaCL, 0.1% SDS and 1% formamide at pH 7.4) with 100 ng of EUB mix of positive control oligonucleotide probes (EUB I, EUB II and EUB III) [22]. The slides were incubated with hybridization buffer with 25 ng of their respective FISH probes for 45 min at 50 ºC, visualized and quantified using a Nikon 80i Eclipse epifluorescence microscope with a Nikon DS-U2 color camera and the NIS-Element imaging software (Nikon, Tokyo, Japan) [22]. Bacteria with uneven distribution or a low overall concentration were enumerated within areas larger than 100 x 100 μm in whole microscopic fields or on the total surface of the fecal cylinder. All counts were performed at a magnification of 1000 times. The conversion of numbers within microscopic areas defined for bacterial concentrations per milliliter was based on the calculation that a sample of 10 μL with a cell concentration of 10^7^ cells/mL has an average of 40 cells per field [22]. The samples were analyzed using the Eub338 mix probe conjugated with fluorescein isothiocyanate (FITC, green signal) at the 5’ end (positive control) [23] and a species-specific probe was conjugated with a single fluorescent carbocyanine molecule (Cy3, red signal). All probes were purchased from MWG Eurofins Operon (Ebersberg, Germany).

### Statistical analysis

Data with non-Gaussian distribution (determined using the Shapiro-Wilk test) were submitted to logarithmic normalization prior to the multiple linear regression analyzes. Differences between the CG and CFG regarding the variables used for characterization were analyzed using Student's t-test, Mann-Whitney test and chi-square test when categorical. Associations among antibiotic use, intestinal inflammation and intestinal microorganisms in the CG and CF groups were evaluated using multiple linear regression analysis adjusted for sex and age. Spearman’s rank correlation coefficients were calculated considering the CG and CFG separately to determine correlations among BMI, intestinal microorganisms and intestinal inflammation. Statistical analysis was performed with the aid of the SPSS program, version 16.0 for Windows^TM^, with a p-value < 0.05 considered indicative of statistical significance.

## Results

### Demographic characteristics, clinical markers, intestinal inflammation and intestinal microbiota

The participants were matched for sex and age, with no difference between the CG and CFG groups (p > 0.05). However, the CFnAB group was composed of younger patients compared to the other groups (Table 1). Delivery was by cesarean birth in the majority of the CFG (73.7%) and vaginal birth in the majority of the CG (52.9%). Based on the supplementation protocols, only one patient in the CFG did not supplement with pancreatic enzymes during the study. Most patients were exclusively breastfed for less than six months (CG: 76.5%; CFG: 73.7%), which explains the frequent use of infant formulas (around 70% in both groups) and the introduction of solid foods before six months of age (Table 1). No significant difference was found between the CG and CFG regarding BMI, which suggests that CF does not affect nutritional status (p = 0.186) (Table 1). In the stratified CFG, however, the CFnAB group had a higher mean BMI compared to the CFAB group (17.32 ± 2.19 kg/m² *vs*. 15.03 ± 0.92 kg/m², p = 0.011), despite being composed mainly of younger patients (Table 1). The majority of patients with CF had the Phe508del mutation (ΔF508) (68.4%); one patient did not have this mutation and the remaining patients were not diagnosed during the study (Table 1). The disease severity score and lung function in the CFG were classified as excellent (88.57 points) and good (80.67%), respectively. About 40% of the patients in the CFG had pulmonary colonization, 15.8% of whom were colonized by *P*. *aeruginosa* (Table 1) and about 25% were colonized by *Staphylococcus aureus* and *Burkholderia cepacia*. These results are in line with the fact that about 50% were using antibiotics (colimicin, azithromycin, tobramycin, cefuroxime, ciprofloxacin, oxacillin, ceftazidime) during the study (Table 1).

**Table 1:** Demographic and clinical markers of the control group (CG), cystic fibrosis group CFG), cystic fibrosis antibiotic therapy (CFAB) and cystic fibrosis absence of antibiotic therapy (CFnAB).

Variables		CG (n = 17)	CFG (n = 19)	p-value^4^	CFAB (n = 10)	CFnAB (n = 9)	p-value^5^
Age^1^^,^^€^		3.00 (0.6–7.0)	4.00 (1.1–9.5)	0.552	6.00 (3.2–10.0)	1.50 (0.8–7.0)	0.447
Sex n (%)^2^^,^^£^	Male	11 (64.7)	10 (52.6)	0.317	03 (30.0)	07 (77.8)	0.051
	Female	06 (35.3)	09 (47.4)		07 (70.0)	02 (22.2)	Fisher
Type of delivery n (%)^2^^,^^£^	Vaginal	09 (52.9)	05 (26.3)	0.237	02 (20.0)	03 (33.3)	0.628
	Cesarean	07 (41.2)	14 (73.7)		08 (80.0)	06 (66.7)	
Breastfeeding n (%)^2^^,^^£^	< 6 months	13 (76.5)	14 (73.7)	**0.003**	07 (70.0)	07 (77.8)	0.701
	≥ 6 months	04 (23.5)	05 (26.3)		03 (30.0)	02 (22.2)	
Infant formula n (%)^2^^,^^£^	No	05 (29.4)	05 (26.3)	0.836	03 (30.0)	02 (22.2)	0.701
	Yes	12 (70.6)	14 (73.7)		07 (70.0)	07 (77.8)	
Starting solid foods n (%)^2^^,^^£^	< 6 months	06 (35.3)	11 (57.9)	0.175	06 (60.0)	05 (55.6)	0.845
	≥ 6 months	11 (64.7)	08 (42.1)		04 (40.0)	04 (44.4)	
BMI (kg/m²)^3^^,^^¥^		17.07 ± 1.38	16.17 ± 2.01	0.186	15.03 ± 0.92	17.32 ± 2.19	**0.011**
Mutation n (%)^2^	Homozygous Phe508del	-	06 (31.6)	-	04 (40.0)	02 (22.2)	-
	Heterozygous Phe508del	-	07 (36.8)	-	04 (40.0)	03 (33.3)	-
	Others		01 (5.3)	-	01 (10.0)	-	-
	No results	-			01 (10.0)	04 (44.4)	-
S-K score (points)		-	88.57 ± 10.88	-	87.50 ± 9.35	89.38 ± 12.37	0.762
FEV_1_ (%)^2^		-	80.67 ± 22.00	-	71.07 ± 25.19	93.48 ± 8.34	0.175
Lung colonization, n (%)^2^	Negative	-	07 (36.8)	-	01 (10.0)	06 (66.7)	-
	*P*. *aeruginosa*	-	03 (15.8)	-	03 (30.0)	-	-
	Others	-	05 (26.3)	-	03 (30.0)	02 (22.2)	-
	No results		04 (21.1)		03 (30.0)	01 (11.1)	-
Antibiotic therapy, n (%)^2^	No	17 (100)	09 (47.4)	-	-	09 (47.4)	-
	Yes	-	10 (52.6)	-	10 (52.6)	-	-

BMI: Body mass index; Shwachman-Kulczychi score: S-K escore. FEV1: Forced Expiratory Volume in the First Second. *P*. *aeruginosa*: *Pseudomonas* aeruginosa.

^1^Values in median and interquartile interval.

^2^Values in frequency and percentage.

^3^Values in mean and standard desviation.

^€^Performed Mann Whitney test.

^£^Performed chi-square test.

^¥^Performed Student's t-test.

p-value^4^: CG *versus* CFG.

p value^5^: CFAB *versus* CFnAB. Significance p-value < 0.05.

Fecal calprotectin (marker of intestinal inflammation) was significantly higher in CFG (median: 40.80 µg/g, IQR: 19.80–87.10 µg/g, p = 0.017) and CFAB group (median: 62.95 µg/g, IQR: 23.80–129.10 µg/g, p = 0.008) compared to the CG (median: 20.15 µg/g, IQR: 16.20–31.00) (Tables 2 and 3). *Bacteroides*, Firmicutes, *E*. *rectale* and *F*. *prausnitzii* were significantly lower (p < 0.05) in the CFG compared to the CG, whereas *C*. *difficile*, *Escherichia coli* (*E*. *coli*) and *P*. *aeruginosa* were significantly higher in the CFG (p < 0.05) (Tables 2 and 3). In the comparison of the two CF groups, only *Bifidobacterium* was significantly lower in the CFAB group compared to the CFnAB group (sex and age adjusted analyses, p = 0.015) (Tables 2 and 4).

**Table 2:** Intestinal inflammation and intestinal microbiota of the control group (CG), cystic fibrosis group CFG), cystic fibrosis antibiotic therapy (CFAB-group) and cystic fibrosis absence of antibiotic therapy (CFnAB-group).

Variables	CG (n = 17)	CFG (n = 19)	CFAB (n = 10)	CFnAB (n = 9)
**Intestinal inflammatory markers**^**#**^				
Fecal calprotectin (µg/g feces)	20.15 (16.20–31.00)	40.80 (19.80–87.10)	62.95 (23.80–129.10)	29.70 (10.40–52.20)
**Intestinal microbiota**^**#**^				
*Bacteroides* (×10^9^/mL feces)	28.30 (19.30–38.00)	15.70 (1.10–24.65)	2.92 (0.00–24.30)	20.00 (2.83–25.00)
*Bifidobacterium* (×10^9^/mL feces)	20.30 (14.65–23.15)	13.00 (5.52–21.20)	5.51 (0.60–17.00)	20.70 (13.00–22.30)
*Veillonella* (×10^9^/mL feces)	0.70 (0.00–11.35)	1.00 (0.00–4.39)	0.87 (0.00–1,77)	1.00 (0.00–7.00)
Firmicutes (×10^9^/mL feces)	9.33 (1.55–12.17)	0.97 (0.77–1.57)	0.98 (0.60–1.53)	0.97 (0.77–1.63)
*Eubacterium rectale* (×10^9^/mL feces)	21.00 (16.00–23.00)	10.70 (3.74–15.15)	12.5 (1.17–18.00)	10.30 (6.30–13.30)
*Clostridium difficile* (×10^9^/ml feces)	0.20 (0.13–0.44)	0.50 (0.34–1.02)	0.54 (0.47–1,07)	0.50 (0.30–0.93)
*Lactobacillus paracasei* (×10^9^/mL feces)	3.17 (2.02–8.47)	2.37 (1.79–5.74)	2.07 (1.40–5.00)	2.77 (2.27–6.47)
*Faecalibacterium prausnitzii* (×10^9^/mL feces)	14.00 (3.92–27.65)	1.00 (0.17–11.50)	0.79 (0.00–4.43)	1.90 (0.33–18.00)
*Escherichia coli* (×10^9^/mL feces)	0.67 (0.40–1.60)	1.47 (1.02–2.67)	1.79 (1.00–2.80)	1.37 (1.27–2.50)
*Pseudomonas aeruginosa* (×10^9^/mL feces)	0.23 (0.15–0.53)	1.13 (0.87–3.92)	0.95 (0.70–1.93)	2.27 (1.10–5.00)

^#^Values presented in median and interquartile interval. Significance values of the comparisons between CG *versus* CF groups (CF, CFAB and CFnAB) and CFAB *versus* CFnAB were represented by the crude “p” values of the multiple linear regression analysis (95% confidence interval) in Tables 3 and 4.

**Table 3:** Multiple linear regression analysis between control group (CG, n = 17) and cystic fibrosis group (CFG, n = 19), between CG (n = 17) and cystic fibrosis antibiotic therapy (CFAB-group), and between CG and cystic fibrosis absence of antibiotic therapy (CFnAB-group).

Variables	Cystic fibrosis group (n = 19)				CFAB (n = 10)				CFnAB (n = 9)			
	Crude β 0 coef.	p-value	Adjusted β 1 coef.	p-value	Crude β 0 coef.	p-value	Adjusted β 1 coef.	p-value	Crude β 0 coef.	p-value	Adjusted β 1 coef.	p-value
**Intestinal inflammatory markers**^**#**^												
Fecal calprotectin (µg/g feces)	-0.384	**0.030**	-0.377	**0.017**	0.545	**0.007**	0.543	**0.008**	0.310	0.161	0.367	0.064
**Intestinal microbiota**^**#**^												
*Bacteroides* (×10^9^/mL feces)	0.435	**0.021**	0.430	**0.024**	-0.495	**0.027**	-0.479	0.051	-0.426	**0.048**	-0.530	**0.015**
*Bifidobacterium* (×10^9^/mL feces)	0.296	0.095	0.275	0.131	-0.529	**0.008**	-0.531	**0.018**	0.008	0.969	-0.008	0.968
*Veillonella* (×10^9^/mL feces)	0.028	0.895	0.031	0.881	-0.087	0.733	-0.143	0.591	0.046	0.862	0.062	0.815
Firmicutes (×10^9^/mL feces)	0.633	**<0.001**	0.573	**<0.001**	-0.622	**0.001**	-0.476	**0.002**	-0.522	**0.009**	-0.549	**0.001**
*Eubacterium rectale* (×10^9^/mL feces)	0.364	**0.041**	0.383	**0.034**	-0.311	0.130	-0.299	0.171	-0.437	**0.033**	-0.490	**0.026**
*Clostridium difficile* (×10^9^/mL feces)	-0.384	**0.027**	-0.419	**0.018**	0.343	0.094	0.440	**0.048**	0.379	0.075	0.347	0.104
*Lactobacillus paracasei* (×10^9^/mL feces)	0.108	0.543	0.130	0.470	-0.184	0.378	-0.340	0.096	-0.020	0.924	-0.008	0.972
*Faecalibacterium prausnitzii* (×10^9^/mL feces)	0.484	**0.009**	0.592	**0.001**	-0.592	**0.005**	-0,737	**0.001**	-0.383	0.087	-0.494	**0.020**
*Escherichia coli* (×10^9^/mL feces)	-0.452	**0.006**	-0.492	**0.002**	0.472	**0.013**	0.570	**0.004**	0.395	**0.046**	0.318	**0.049**
*Pseudomonas aeruginosa* (×10^9^/mL feces)	-0.727	**<0.001**	-0.728	**<0.001**	0.686	**<0.001**	0.607	**0.001**	0.785	**<0.001**	0.817	**<0.001**

β coef.: beta coefficient; β 0 coefficient: crude values; β 1 coefficient: values adjusted for confounding variables (sex and age).

^#^ Values ​​were normalized by log_10_ transformation.

“p-value” values were derived from the multiple linear regression analysis (95% confidence interval).

Boldface data indicate statistical significance (p-value < 0.05).

**Table 4:** Multiple linear regression analysis between cystic fibrosis antibiotic therapy (CFAB-group, n = 10) and cystic fibrosis absence of antibiotic therapy (CFnAB-group, n = 9).

Variables	Crude β 0 coef.	p-value	Adjusted β 1 coef.	p-value
**Intestinal inflammatory markers** ^**#**^				
Fecal calprotectin (µg/g feces)	-0.321	0.181	-0.223	0.320
**Intestinal microbiota** ^**#**^				
*Bacteroides* (×10^9^/mL feces)	0.076	0.796	-0.032	0.932
*Bifidobacterium* (×10^9^/mL feces)	0.532	**0.028**	0.725	**0.015**
*Veillonella* (×10^9^/mL feces)	0.153	0.617	0.368	0.284
Firmicutes (×10^9^/mL feces)	0.271	0.276	0.234	0.405
*Eubacterium rectale* (×10^9^/mL feces)	-0.013	0.962	-0.160	0.656
*Clostridium difficile* (×10^9^/mL feces)	0.108	0.669	-0.019	0.948
*Lactobacillus paracasei* (×10^9^/mL feces)	0.188	0.454	0.185	0.543
*Faecalibacterium prausnitzii* (×10^9^/mL feces)	0.224	0.442	0.417	0.119
*Escherichia coli* (×10^9^/mL feces)	-0.138	0.572	-0.169	0.544
*Pseudomonas aeruginosa* (×10^9^/mL feces)	0.291	0.241	0.375	0.202

β coef.: beta coefficient; β 0 coefficient: crude values; β 1 coefficient: values adjusted for confounding variables (sex and age).

^# ^Values ​​were normalized by log_10_ transformation.

“p-values” were derived from the multiple linear regression analysis (95% confidence interval).

Boldface data indicate statistical significance (p-value < 0.05).

### Associations between the use of antibiotics and both fecal calprotectin and intestinal microorganisms

In the multiple linear regression analysis of the use of antibiotics adjusted for sex and age, fecal calprotectin was significantly higher in the CFAB group (median: 62.95 μg/g feces, IQR: 21.80–136.63) compared to the CG (median: 20.15 μg/g feces, IQR: 16.20–31.00, p = 0.007) (Tables 2 and 3), whereas no significant difference was found between the CFAB and CFnAB groups (sex and age adjusted analyses, p = 0.181) (Tables 2, 3 and 4).

With regard to intestinal microorganisms, *E*. *rectale* and Firmicutes were significantly higher in the GC compared to both the CFAB (*E*. *rectale*: p = 0.027; Firmicutes: p = 0.002) and CFnAB (*E*. *rectale*: p = 0.003; Firmicutes: p = 0.004) groups, with no significant difference between the two CF groups (Tables 2 and 3). *Bifidobacterium* was significantly lower in the CG compared to the CFAB group (p = 0.011), but no significant difference was found in comparison to the CFnAB group (p = 1.000) (Tables 2 and 3). *Bifidobacterium* was the only microorganism analyzed that was significantly lower in the CFAB group than the CFnAB group (p = 0.010) (Tables 2 and 4). *C*. *difficile* and *P*. *aeruginosa* were lower in the CG compared to both the CFAB (*C*. *difficile*: p = 0.001; *P*. *aeruginosa*: p = 0.001) and CFnAB (*C*. *difficile*: p = 0.033; *P*. *aeruginosa*: p < 0.001) groups, with no significant difference between the two CF groups (Tables 2–4).

### Correlations between intestinal microorganisms and both BMI and fecal calprotectin

**Fig 1:**
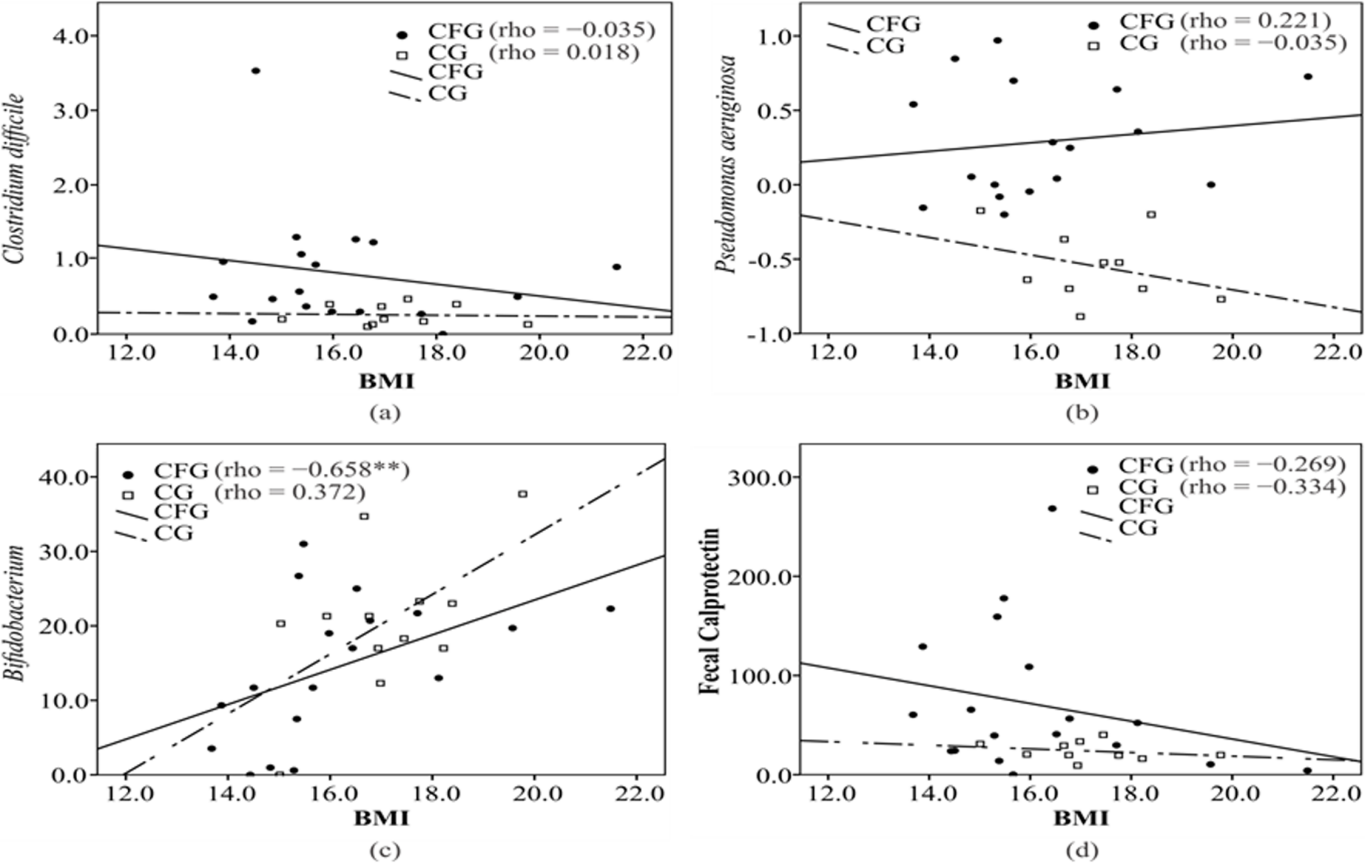
Correlation between body mass index (BMI) and intestinal microorganisms.Cystic fibrosis group (CFG), Control group (CG).*p <0.05. BMI: Body mass index. CFG: Cystic fibrosis group. CG: Control group.

**Fig 2:**
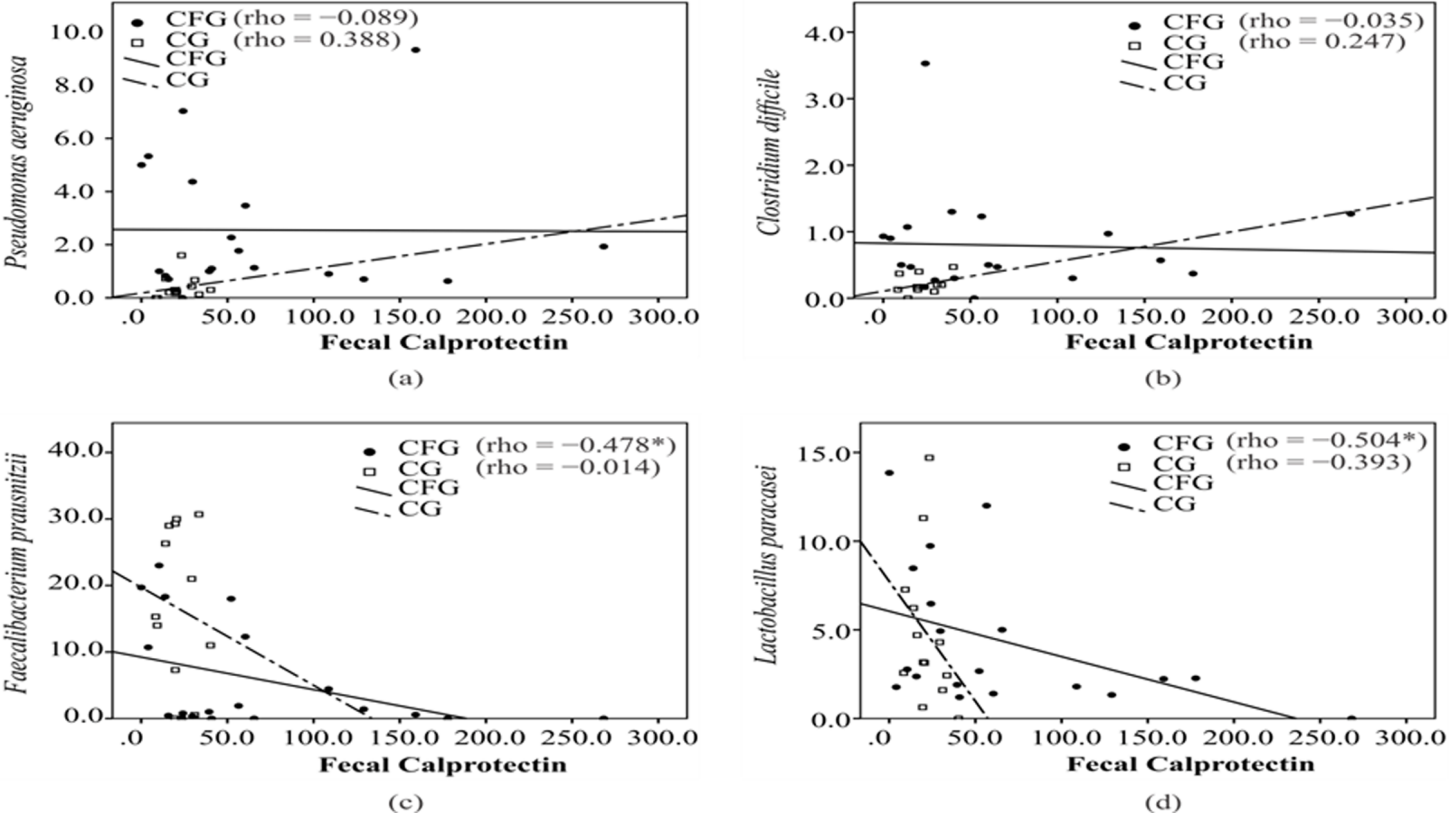
Correlation between fecal calprotectin and intestinal microorganisms in cystic fibrosis group (CFC) and control group (CG). * p <0.05. CFG: Cystic fibrosis group. CG: Control group. Spearman’s correlation, *p < 0.05.

A positive correlation was found between BMI and *Bifidobacterium* in the CFG (CFG: rho = 0.658, p = 0.003; CG: rho = 0.372, p = 0.234) (Fig 1C), whereas no significant correlations were found between BMI and other microorganisms (Fig 1 and S2 and S4 Tables). Negative correlations were found between fecal calprotectin and both *F*. *prausnitzii* (CFG: rho = -0.478, p = 0.039; CG: rho = -0.014, p = 0.964) (Fig 2C) and *L*. *paracasei* (CFG: rho = -0.504, p = 0.028; CG = rho = -0.393, p = 0.184) (Fig 2D), whereas no significant correlations were found for other microorganisms. A positive correlation was found between Bacteroides and *L*. *paracasei* (CFG: rho = 0.503, p = 0.028; CG: rho = -0.184, p = 0.480) (supplementary material) and a negative correlation was found between *Veillonella* and *E*. *rectale* in the CFG (CFG: rho = , p = 0.027; CG: rho = 0.078, p = 0.766) (S1 and S3 Tables).

## Discussion

The literature reports intestinal abnormalities in the microbiota of patients with CF [6, 12, 24]. However, it is unclear whether this altered microbiota is the consequence of the disease or antibiotic therapy and the implications of these changes remain unknown. The present study shows a different pattern of bacterial species.

The patients in the CFG had a similar BMI to the volunteers in the CG as well as adequate lung function and an “excellent” disease severity score (≥ 86 points), although the majority had the ΔF508 mutation, which is associated with the impairment of lung function and a consequent worsening of nutritional status [25]. Pulmonary colonization by bacteria was found in about 40% of the CFG. Moreover, one of the patients did not present pulmonary colonization by *P*. *aeruginosa* at the collection time, but the bacterium was present in a large amount in the intestinal microbiota. About three months after the study, the patient exhibited pulmonary colonization by *P*. *aeruginosa*, which suggests that intestinal colonization may precede pulmonary colonization [26]. However, longitudinal studies are needed to confirm this assumption.

The positive correlation found between BMI and *Bifidobacterium* in the CFG shows that an improvement in nutritional status may be related to an increase of *Bifidobacterium*, which, together with *Faecalibacterium* and *E*. *rectale*, is regarded as a marker of healthy intestinal microbiota [15, 27]. Decreased quantities of these bacteria can affect the nutrition of enterocytes and, consequently, the maintenance of intestinal health [27, 28]. After weaning, *Bifidobacterium* gradually decreases and *Bacteroides* and *Eubacterium* become predominant, but it is still considered a determinant of adult intestinal health [27]. *Bifidobacterium* was the only microorganism that was significantly decreased in the CFAB group compared to the CFnAB group, which may be related to the use of antibiotics.

With regard to breastfeeding, approximately 70% of both groups (CG and CFG) were exclusively breastfed for less than six months and the CFG had lower values of the phylum Firmicutes. Lactate-producing bacteria, such as *Lactobacillus*, and those that use lactate, such as bacteria belonging to the genera *Veillonella* and *Faecalibacterium* and the phylum Firmicutes, provide the host with important short-chain fatty acid metabolites, which are related to protection against intestinal inflammation [6, 29]. The intestinal microbiome develops throughout childhood, during which the assemblage is characterized by an increase in colonization by *Bifidobacteria* and microorganisms belonging to the phyla Firmicutes and Bacteroidetes as well as a reduction in *Enterobacteria*. Thus, any alteration in this colonization process leads to a change in the composition of the microbiota [30].

Fecal calprotectin is considered a good marker of intestinal inflammation [31] and was increased in the CFG (approximately 47% of the group had concentrations higher than 50 µg/g of feces). This is in agreement with data described in the literature [6, 12]. The negative correlation between fecal calprotectin and both *F*. *prausnitzii* and *L*. *paracasei* suggest that intestinal inflammation may be related to a reduction in these bacteria. *F*. *prausnitzii* is a butyrate-producing bacterium and the decrease in the production of this substance can exert an influence on both the nutritional status of enterocytes and luminal pH, consequently affecting the maintenance of intestinal health [6]. *Lactobacillus* spp. are lactic acid-producing bacteria, which is reported to be associated with the prevention of respiratory infections [32]. In the analysis of the association between fecal calprotectin and the use of antibiotics, the influence of antibiotic therapy on the development of intestinal inflammation in CF was not clear. Although a difference was found between the CF groups, it was non-significant, which may be a consequence of the sample size.

In the present study, butyrate-producing (*E*. *rectale* and *F*. *prausnitzii*), lactic acid-producing (*L*. *paracasei* and *Bifidobacterium* spp.) and acetate-producing (*Bacteroides*) microorganisms were significantly lower and *E*. *coli*, *C*. *difficile* and *P*. *aeruginosa* were higher in the CFG compared to the CG, which is in agreement with data reported in the literature [6, 12, 27]. In contrast, a previous study found no difference regarding *Veillonella* bacteria in the patients with cystic fibrosis [33].

The negative correlation between *Veillonella* and *E*. *rectale* and the positive correlation between *Bacteroides* and *L*. *paracasei* demonstrate a relationship among these microorganisms.

The microorganisms *Bacteroides*, *E*. *rectale*, *F*. *prausnitzii*, *Bifidobacterium* and Firmicutes were significantly lower in the CFAB group compared to the CG, whereas *E*. *coli*, *C*. *difficile* and *P*. *aeruginosa* were significantly higher in the CFAB group compared to the CG. It should be noted that these changes in the intestinal microbiota were also found in the CFnAB group.

Burk (2017) [24] found alterations in the intestinal microbiota of adults with CF, with an increase in the phylum Firmicutes and a decrease in *Bacteriodes*, which differs from the present findings. In contrast, Bruzzese (2014) [6] found similar results to those of the present investigation in a study involving children with CF. This difference may be due to the methods employed as well as the difference in age, which consequently exerts an influence on the composition of the intestinal microbiota.

As previously reported in a study by Hoffman (2014) [34], *E*. *coli* was significantly increased in the CFG. In a study involving patients with CF aged 10 to 22 years, Miragoli (2017) [12] found an increase in *E*. *coli* in the group with CF, but the difference was non-significant in comparison to the control group. The increase in *E*. *coli* has been related to dysbiosis and the abundance of *E*. *coli* in patients with CF may be due to the decreased motility and/or accumulation of mucus [35]. Evidence suggests that changes in the intestinal lumen during an inflammatory process may increase the ability of *E*. *coli* to colonize the intestinal mucosa and induce further inflammation [34]. Hoffman (2017) [34] suggests that *E*. *coli* may serve as a marker and cause of gastrointestinal tract dysfunction and disease in this population.

*C*. *difficile* is considered the major entero-pathogen of antibiotic-associated diarrhea and is responsible for cases of pseudomembranous colitis [36, 37]. Indeed, this bacterium was found to be increased in the CFG and CFAB group in the present study.

*P*. *aeruginosa* is considered the most aggressive bacterium with regard to pulmonary function in patients with CF. However, the occurrence and influence of this bacterium in the intestinal microbiota of this population has not previously been studied. One study found that *P*. *aeruginosa* was the most frequent species identified in the feces of patients with irritable bowel syndrome and may be involved in the pathophysiology of the disease [38], indicating that it may also be related to intestinal dysbiosis. The increase in *P*. *aeruginosa* in the intestinal microbiota and the fact that it preceded pulmonary colonization should be studied further to enable a better understanding of the mechanisms involved as well as the harm that it can cause.

The microorganisms evaluated herein were selected based on their influence on the maintenance of intestinal health and the relationship with CF reported in the literature [6, 8, 12, 34]. Therefore, the failure to analyze the microbiota population as a whole should be considered a limitation of the present study. Other limitations are cross-sectional design, which does not enable inferences regarding cause and effect, and the small sample size. However, the results of the present study confirm the presence of intestinal inflammation in patients with CF and suggest that the disease may be related to changes in the colonization of some microorganisms. The frequent use of antibiotics does not appear to be closely related to the change in intestinal microbiota, but further studies are needed to reach such conclusions.

## Supporting information

S1 Table Spearman’s rank correlations between intestinal microorganisms in the cystic fibrosis group (n = 19). *P*. *aeruginosa*: *Pseudomonas aeruginosa*. *E*. *rectale*: *Eubacterium rectale*. *F*. *prausnitzii*: *Faecalibacterium prausnitzii*. *L*. *paracasei*: *Lactobacillus paracasei*. *E*. *coli*: *Escherichia coli*. *C*. *difficile*: *Clostridium difficile*. * Correlation is significant at the 0.05 level (2-tailed).
(DOCX)

S2 Table Spearman’s rank correlations between intestinal microorganisms and fecal calprotectin and body mass index in the cystic fibrosis group. *P*.*aeruginosa*: *Pseudomonas aeruginosa*. *E rectale*: *Eubacterium rectale*. *F*. *prausnitzii*: *Faecalibacterium prausnitzii*. *L*. *paracasei*: *Lactobacillus paracasei*. *E*. *coli*: *Escherichia coli*. *C*. *difficile*: *Clostridium difficile*. F. Cal: Fecal Calprotectin (n = 19). BMI: body mass index (n = 18). *Correlation is significant at the 0.05 level (2-tailed). **Correlation is significant at the 0.01 level (2-tailed).
(DOCX)

S3 Table Spearman’s rank correlations between intestinal microorganisms in the control group (CG). *P*.*aeruginosa*: *Pseudomonas aeruginosa*. *E rectale*: *Eubacterium rectale*. *F*. *prausnitzii*: *Faecalibacterium prausnitzii*. *L*. *paracasei*: *Lactobacillus paracasei*. *E*. *coli*: *Escherichia coli*. *C*. *difficile*: *Clostridium difficile*. (n = 16)^1^ - (n = 15)^2^. *Correlation is significant at the 0.05 level (2-tailed). **Correlation is significant at the 0.01 level (2-tailed).
(DOCX)

S4 Table Spearman’s rank correlations between intestinal microorganisms and fecal calprotectin (F. Cal.) and body mass index (BMI) in the control group (n = 19). *P*.*aeruginosa*: *Pseudomonas aeruginosa*. *E rectale*: *Eubacterium rectale*. *F*. *prausnitzii*: *Faecalibacterium prausnitzii*. *L*. *paracasei*: *Lactobacillus paracasei*. *E*. *coli*: *Escherichia coli*. *C*. *difficile*: *Clostridium difficile*. Fecal calprotectin: (n = 10)^1^ (n = 12)^2^. BMI: (n = 10)^1^ (n = 11)^3^. *Correlation is significant at the 0.05 level (2-tailed). **Correlation is significant at the 0.01 level (2-tailed).
(DOCX)
