# Supplementary material for: Altered intestinal microbiota composition, antibiotic therapy and intestinal inflammation in children and adolescents with cystic fibrosis
Source: PLoS One. 2018 Jun 22;13(6):e0198457. doi: 10.1371/journal.pone.0198457 (PMC6014676; doi:10.1371/journal.pone.0198457)
Supplement: S1 Table — P. aeruginosa: Pseudomonas aeruginosa. E. rectale: Eubacterium rectale. F. prausnitzii: Faecalibacterium prausnitzii. L. paracasei: Lactobacillus paracasei. E. coli: Escherichia coli. C. difficile: Clostridium difficile. * Correlation is significant at the 0.05 level (2-tailed). (DOCX) [file pone.0198457.s001.docx]

S1 Table. Spearman’s rank correlations between intestinal microorganisms in the cystic fibrosis group.

|  |  | ***P.***  ***aeruginosa*** | **Firmicutes** | ***Veillonella*** | ***Bacteroides*** | ***E.***  ***rectale*** | ***F.***  ***prausnitzii*** | ***Bifidobacterium*** | ***L.***  ***paracasei*** | ***E.***  ***coli*** | ***C. difficile*** |
| --- | --- | --- | --- | --- | --- | --- | --- | --- | --- | --- | --- |
| ***P. aeruginosa*** | (rho) | 1.000 | .249 | -.083 | .098 | -.248 | .222 | .053 | .021 | .208 | .258 |
| (n = 19) | (p) | . | .304 | .734 | .689 | .305 | .360 | .829 | .932 | .393 | .285 |
| **Firmicutes** | (rho) |  | 1.000 | -.057 | .280 | .119 | .330 | -.186 | -.042 | -.440 | -.263 |
| (n = 19) | (p) |  | . | .816 | .245 | .627 | .168 | .445 | .864 | .059 | .276 |
| ***Veillonella*** | (rho) |  |  | 1.000 | -.220 | -.507^*^ | -.281 | -.206 | .190 | .044 | -.140 |
| (n = 19) | (p) |  |  | . | .365 | **.027** | .243 | .399 | .436 | .858 | .567 |
| ***Bacteroides*** | (rho) |  |  |  | 1.000 | .394 | .455 | .191 | .503^*^ | -.130 | -.121 |
| (n = 19) | (p) |  |  |  | . | .095 | .050 | .433 | **.028** | .595 | .621 |
| ***E. rectale*** | (rho) |  |  |  |  | 1.000 | .201 | -.005 | .008 | .019 | -.234 |
| (n = 19) | (p) |  |  |  |  | . | .409 | .984 | .974 | .939 | .335 |
| ***F. prausnitzii*** | (rho) |  |  |  |  |  | 1.000 | .139 | .214 | -.174 | .214 |
| (n = 19) | (p) |  |  |  |  |  | . | .570 | .379 | .475 | .378 |
| ***Bifidobacterium*** | (rho) |  |  |  |  |  |  | 1.000 | -.062 | .165 | -.039 |
| (n = 19) | (p) |  |  |  |  |  |  | . | .800 | .499 | .874 |
| ***L. paracasei*** | (rho) |  |  |  |  |  |  |  | 1.000 | -.031 | -.002 |
| (n = 19) | (p) |  |  |  |  |  |  |  | . | .901 | .994 |
| ***E. coli*** | (rho) |  |  |  |  |  |  |  |  | 1.000 | .108 |
| (n = 19) | (p) |  |  |  |  |  |  |  |  | . | .661 |
| ***C. difficile*** | (rho) |  |  |  |  |  |  |  |  |  | 1.000 |

*P. aeruginosa:* *Pseudomonas aeruginosa;* *E. rectale: Eubacterium rectale; F. prausnitzii: Faecalibacterium prausnitzii; L. paracasei: Lactobacillus paracasei; E. coli: Escherichia coli; C. difficile: Clostridium difficile.* * Correlation is significant at the 0.05 level (2-tailed).
