# Supplementary material for: Altered intestinal microbiota composition, antibiotic therapy and intestinal inflammation in children and adolescents with cystic fibrosis
Source: PLoS One. 2018 Jun 22;13(6):e0198457. doi: 10.1371/journal.pone.0198457 (PMC6014676; doi:10.1371/journal.pone.0198457)
Supplement: S2 Table — P.aeruginosa: Pseudomonas aeruginosa. E rectale: Eubacterium rectale. F. prausnitzii: Faecalibacterium prausnitzii. L. paracasei: Lactobacillus paracasei. E. coli: Escherichia coli. C. difficile: Clostridium difficile. F. Cal: Fecal Calprotectin (n = 19). BMI: body mass index (n = 18). *Correlation is significant at the 0.05 level (2-tailed). **Correlation is significant at the 0.01 level (2-tailed). (DOCX) [file pone.0198457.s002.docx]

S2 Table. Spearman’s rank correlations between intestinal microorganisms and fecal calprotectin and body mass index in the cystic fibrosis group.

|  |  | **F. Cal.** | **BMI** | ***P. aeruginosa*** | **Firmicutes** | ***Veillonella*** | ***Bacteroides*** | ***E. rectale*** | ***F. prausnitzii*** | ***Bifidobacterium*** | ***L. paracasei*** | ***E.***  ***coli*** | ***C. difficile*** |
| --- | --- | --- | --- | --- | --- | --- | --- | --- | --- | --- | --- | --- | --- |
| **F. Cal.** | (rho) | 1.000 | -.269 | -.089 | .056 | .305 | -.292 | -.247 | -.478^*^ | -.020 | -.504^*^ | .036 | -.035 |
| (n = 19) | (p) | . | .280 | .718 | .819 | .205 | .225 | .308 | .**039** | .935 | **.028** | .884 | .886 |
| **BMI** | (rho) | -.269 | 1.000 | .221 | .011 | .089 | .239 | -.310 | .260 | .658^**^ | -.015 | .215 | -.218 |
| (n = 18) | (p) | .280 | . | .378 | .964 | .724 | .340 | .210 | .298 | **.003** | .951 | .392 | .385 |

*P. aeruginosa:* *Pseudomonas aeruginosa;* *E. rectale: Eubacterium rectale; F. prausnitzii: Faecalibacterium prausnitzii; L. paracasei: Lactobacillus paracasei; E. coli: Escherichia coli; C. difficile: Clostridium difficile.*

F. Cal: Fecal Calprotectin (n = 19). BMI: body mass index (n = 18).

*Correlation is significant at the 0.05 level (2-tailed). **Correlation is significant at the 0.01 level (2-tailed).
