# Supplementary material for: Altered intestinal microbiota composition, antibiotic therapy and intestinal inflammation in children and adolescents with cystic fibrosis
Source: PLoS One. 2018 Jun 22;13(6):e0198457. doi: 10.1371/journal.pone.0198457 (PMC6014676; doi:10.1371/journal.pone.0198457)
Supplement: S3 Table — P.aeruginosa: Pseudomonas aeruginosa. E rectale: Eubacterium rectale. F. prausnitzii: Faecalibacterium prausnitzii. L. paracasei: Lactobacillus paracasei. E. coli: Escherichia coli. C. difficile: Clostridium difficile. (n = 16)1 - (n = 15)2. *Correlation is significant at the 0.05 level (2-tailed). **Correlation is significant at the 0.01 level (2-tailed). (DOCX) [file pone.0198457.s003.docx]

S3 Table. Spearman’s rank correlations between intestinal microorganisms in the control group (CG).

|  |  | ***P. aeruginosa*** | **Firmicutes** | ***Veillonella*** | ***Bacteroides*** | ***E.***  ***rectale*** | ***F. prausnitzii*** | ***Bifidobacterium*** | ***L. paracasei*** | ***E.***  ***coli*** | ***C. difficile*** |
| --- | --- | --- | --- | --- | --- | --- | --- | --- | --- | --- | --- |
| ***P .aeruginosa*** | (rho) | 1.000 | -.347^1^ | .305 | .238 | .354 | -.294 | -.137 | .091 | -.566^*^ | -.306^1^ |
| (n = 17) | (p) | . | .187 | .234 | .358 | .163 | .253 | .601 | .729 | **.018** | .249 |
| **Firmicutes** | (rho) |  | 1.000 | .385 | .185 | .186 | .000 | .478 | -.481 | .530^*^ | .165**^2^** |
| (n = 16) | (p) |  | . | .141 | .494 | .491 | 1.000 | .061 | .059 | **.035** | .556 |
| ***Veillonella*** | (rho) |  |  | 1.000 | .039 | .078 | -.512^*^ | .310 | -.113 | .414 | .178**^1^** |
| (n = 17) | (p) |  |  | . | .882 | .766 | **.036** | .226 | .665 | .098 | .509 |
| ***Bacteroides*** | (rho) |  |  |  | 1.000 | .674^**^ | .165 | .107 | -.184 | .174 | -.108**^1^** |
| (n = 17) | (p) |  |  |  | . | .**003** | .527 | .682 | .480 | .503 | .691**^1^** |
| ***E.rectale*** | (rho) |  |  |  |  | 1.000 | .219 | .041 | -.111 | .033 | -.268**^1^** |
| (n = 17) | (p) |  |  |  |  | . | .398 | .877 | .673 | .899 | .315 |
| ***F. prausnitzii*** | (rho) |  |  |  |  |  | 1.000 | -.122 | .143 | -.060 | -.053**^1^** |
| (n = 17) | (p) |  |  |  |  |  | . | .640 | .585 | .818 | .844 |
| ***Bifidobacterium*** | (rho) |  |  |  |  |  |  | 1.000 | -.149 | .346 | -.072**^1^** |
| (n = 17) | (p) |  |  |  |  |  |  | . | .569 | .173 | .792 |
| ***L. paracasei*** | (rho) |  |  |  |  |  |  |  | 1.000 | -.033 | -.060**^1^** |
| (n = 17) | (p) |  |  |  |  |  |  |  | . | .900 | .826 |
| ***E. coli*** | (rho) |  |  |  |  |  |  |  |  | 1.000 | .353**^1^** |
| (n = 17) | (p) |  |  |  |  |  |  |  |  | . | .180 |
| ***C .difficile*** | (rho) |  |  |  |  |  |  |  |  |  | 1.000 |
| (n = 17) |  |  |  |  |  |  |  |  |  |  | . |

*P. aeruginosa:* *Pseudomonas aeruginosa;* *E. rectale: Eubacterium rectale; F. prausnitzii: Faecalibacterium prausnitzii; L. paracasei: Lactobacillus paracasei; E. coli: Escherichia coli; C. difficile: Clostridium difficile.*

(n = 16)^1^ - (n = 15)^2^ *Correlation is significant at the 0.05 level (2-tailed). **Correlation is significant at the 0.01 level (2-tailed).
