# Supplementary material for: Altered intestinal microbiota composition, antibiotic therapy and intestinal inflammation in children and adolescents with cystic fibrosis
Source: PLoS One. 2018 Jun 22;13(6):e0198457. doi: 10.1371/journal.pone.0198457 (PMC6014676; doi:10.1371/journal.pone.0198457)
Supplement: S4 Table — P.aeruginosa: Pseudomonas aeruginosa. E rectale: Eubacterium rectale. F. prausnitzii: Faecalibacterium prausnitzii. L. paracasei: Lactobacillus paracasei. E. coli: Escherichia coli. C. difficile: Clostridium difficile. Fecal calprotectin: (n = 10)1 (n = 12)2. BMI: (n = 10)1 (n = 11)3. *Correlation is significant at the 0.05 level (2-tailed). **Correlation is significant at the 0.01 level (2-tailed). (DOCX) [file pone.0198457.s004.docx]

S4 Table. Spearman’s rank correlations between intestinal microorganisms and fecal calprotectin (F. Cal.) and body mass index (BMI) in the control group.

|  |  | **F. Cal.** | **BMI** | ***P. aeruginosa*** | **Firmicutes** | ***Veillonella*** | ***Bacteroides*** | ***E. rectale*** | ***F. prausnitzii*** | ***Bifidobacterium*** | ***L. paracasei*** | ***E.***  ***coli*** | ***C. difficile*** |
| --- | --- | --- | --- | --- | --- | --- | --- | --- | --- | --- | --- | --- | --- |
| **F. Cal.^a^** | (rho) | 1.000 | -.334**^1^** | .388 | .112**^2^** | .084 | -.064 | .122 | -.014 | -.010 | -.393 | -.308 | .247 |
| (n = 13) | (p) | . | .345 | .190 | .729 | .785 | .836 | .692 | .964 | .975 | .184 | .306 | .416 |
| **BMI ^a^** | (rho) | -.334**^1^** | 1.000 | -.035 | .336**^3^** | .703^*^ | -.007 | -.120 | -.316 | .372 | -.084 | .284 | .018 |
| (n = 12) | (p) | .345 | . | .914 | .312 | **.011** | .983 | .711 | .317 | .234 | .795 | .372 | .957 |

*P. aeruginosa:* *Pseudomonas aeruginosa;* *E. rectale: Eubacterium rectale; F. prausnitzii: Faecalibacterium prausnitzii; L. paracasei: Lactobacillus paracasei; E. coli: Escherichia coli; C. difficile: Clostridium difficile.*

**^a^** Sample for the Spearman’s rank correlations analysis were reduced to fecal calprotectin and BMI.

Fecal calprotectin: (n = 10)^1^ and (n = 12)^2^. BMI: (n = 10)^1^ and (n = 11)^3^. *Correlation is significant at the 0.05 level (2-tailed).
